# Twelve Tips for Preparing a Surgical Discharge Summary: Enabling a Safe Discharge

**DOI:** 10.15694/mep.2019.000039.1

**Published:** 2019-03-05

**Authors:** Stephanie Lumpkin, Ian Kratzke, Meredith Duke, Nicole Chaumont

**Affiliations:** 1 University of North Carolina at Chapel Hill; 2 Vanderbilt University

**Keywords:** Quality Improvement, Resident Education, Graduate Medical Education, Transitions of Care, Postsurgical readmissions

## Abstract

This article was migrated. The article was marked as recommended.

The surgical discharge summary allows the perioperative care team to summarize a recent hospitalization and relay important information to a variety of invested parties including other healthcare providers, outpatient caregivers, and the surgical patient. The inpatient care team can promote a smooth transition of care and empower outpatient providers and the patient to foster a confident progression through recovery. We describe twelve tips for a streamlined, successful discharge summary geared towards the surgical intern. A successful surgical discharge summary begins with patient and caregiver collaboration, communication and teamwork, and culminates with concise documentation. These tips reflect a review of the current literature and rely on the clinical expertise of an interdisciplinary surgical team. Our aim is to empower surgical educators and trainees to understand the complexity of discharge planning, and to improve the efficiency with which it can be completed and the quality of the discharge process at their training hospitals.

## Introduction

Surgical trainees are often reminded that “discharge planning starts at the time of admission.” This declaration, however, rarely comes with a comprehensive lesson on what discharge planning actually entails, who it involves, how to do it well and why it is so important.

Within the last ten years, the Center for Medicare and Medicaid Services has begun to shift toward value-based payment, which uses readmission rates greater than expected as a quality metric. This shift in healthcare service reimbursement, has fueled investigation into causes for readmissions and methods for preventing and reducing them ( *Readmissions Reduction Program (HRRP) - Centers for Medicare & Medicaid Services*, 2016). In a systematic review of interventions aimed at reducing 30-day readmissions in medical patients, Hansen et. al couldn’t identify one intervention that independently reduced readmissions rates, but did conclude that proper discharge planning includes bridging the pre-discharge and post-discharge needs of a patient. The group also acknowledged that the most comprehensive pathways aimed at reducing readmissions included the following components: pre-discharge interventions, post-discharge interventions and bridging interventions ( Hansen et al. 2011). Specifically, in the pre-discharge interventions, optimal discharge planning includes patient education, medication reconciliation, disposition planning and scheduling of follow-up care for patients, which we have included in our 12 tips for preparing a surgical discharge summary.

The discharge summary is the only document relaying the ongoing patient care plan to the post-hospital patient care team, whether that be at home, in a nursing facility or a rehabilitation center ( Kripalani et al. 2007). Other groups have focused on the impact of poor care coordination on adverse events affecting patients after discharge from the hospital. At a multisite teaching hospital in Canada, Forster et. al found that 23% of patients suffered an adverse event within 30 days of discharge. Roughly half of the adverse events that occurred after discharge were felt to be preventable, most commonly due to adverse drug events (72%) and therapeutic errors (16%) ( Forster et al. 2004).

The role of the surgical intern in discharge planning is expansive, and includes everything from patient-directed goal-setting, disposition coordination, patient education, and ultimately clear and concise documentation ( Card et al. 2014; Black & Colford 2017; Agency for Healthcare Research and Quality 2017). This comprehensive process, which requires a multi-disciplinary team with varying interests and schedules, can be difficult to coordinate and time-consuming. For a surgical intern who is already tasked with learning complex perioperative care, mastering surgical technique, and addressing the continuous and fluid needs of the myriad of parties relying on them from patients, nurses, case managers, pharmacists, therapists, resident colleagues, and attending physicians, this can seem daunting, if not impossible. Our anecdotal experience is reflected in the literature, which states that the pressures of discharging a patient on time, in the setting of already limited time to complete administrative tasks, can lead to burnout and career dissatisfaction ( Shanafelt et al. 2009). The following twelve tips will arm the surgical intern with a solid understanding of the components of a successful discharge summary, and a useful plan on how to complete it efficiently.

## Twelve Tips

**1**. ***Understand the goal**.* The universal goal of the perioperative patient care team and the patient is a safe discharge home with resumption of regular activities and improved quality of life.

a. For the patient and their family, this means moving toward recovery, taking over responsibility for their continued care (e.g. medication administration, wound care, physical activity, monitoring for complications and adverse events) and preparing to reengage in work or other activities. While a sign of progress, the transition home can be an anxiety-provoking time for patients as they leave an environment with perceived safety related to frequent physical examination and proximity to their medical team, and enter one without daily support or observation ( Horstman et al. 2017).

b. Ancillary care providers (e.g. PT, OT, Home Health RNs, etc.) have often been arranged to help with various parts of post-discharge care needs, but will need explicit, detailed instructions on what was done and what is needed. Without explicit instructions, it is difficult to know what activity level is recommended, how to care for surgical wounds and drains, and how to manage enteral or parenteral nutrition safely.

c. For primary care providers, this is also a time of transition, when they will be expected to resume responsibility over the patient’s care, continue managing old and new medications and monitor for changes that should prompt surgical reevaluation.

When preparing a discharge summary, consider these various shared goals and expectations.

**2**. ***Determine the patient’s baseline health status and social situation**.* Most surgical patients have passed an initial screening process confirming that they are healthy enough to undergo surgery with a reasonable risk profile. This, however, does not replace the need for a baseline history and physical exam on admission for surgery. This should include confirmation of past and ongoing medical issues, as well as a clear picture of their current social situation, including available caregivers and support, occupational requirements that might be influenced by physical restrictions or other factors in their post-operative recovery. Knowing and accurately documenting a patient’s past medical history, surgical history, preoperative medications, and social history is critical for anticipating the potential obstacles in their post-operative course, which will help you care for them acutely, but will also help you anticipate their risk of post-discharge complications and unplanned, high resource healthcare utilization, such as readmissions and emergency department visits.

***3. Anticipate and start early.*** While all patients are different, a good clinician can estimate a normal course of recovery and what is routinely expected after surgery based on their knowledge acquired in Tip #2 and their understanding of the specific surgical procedure the patient has undergone. For our surgical patients, we find that anticipating specific patient needs as early as postoperative day zero, such as ostomy care, wound care needs, TPN, and drain care, can help reduce the frantic rush of discharging patients in a timely manner since most of these things will require time, sometimes days, to coordinate.

As a surgical resident, learning about conditions and surgical treatments in this manner will help with setting clear expectations for patients and establishing a professional and confident rapport. Patients rely on this information on a daily basis to assess their own recovery, and will continue to do so when they are discharged. It is very important to set forth clear expectations, as it has been established that patient expectations are very heavily correlated with patient outcomes after surgery ( Halawi et al. 2015; Auer et al. 2016). From our own qualitative interviews, acknowledging normal changes after surgery can alleviate a lot of stress and anxiety amongst patients. Just as it is our duty to anticipate what types of complications may arise after surgery during a hospitalization, we should do the same for the time period after discharge, attempt to prevent them, and share these concerns with the patient.

**4. *Familiarize yourself with the surgical course and patient’s current status***. Knowing your patient well from the beginning of their hospital course is the best way to prepare for a successful discharge at the end. Once you have established the patient’s medical and social starting points, you can better assess their recovery progress and final goals.

a. What surgery did they have and why did they have it? If this information is not available through personal experience in the operating room or on review of the operative report, engaging your senior residents and attendings can be very helpful. In addition, it will show your interest and enthusiasm for learning, and your commitment to a comprehensive understanding of patient’s pathology, treatment, and experience. This will empower you to provide the patient the best care.

b. What should their hospital course look like? How long are they expected to be here? What goals do they need to achieve to be deemed fit for discharge? As an intern you might not know how a patient is expected to progress through their hospital stay at the beginning. Until you learn this, ask for guidance on what to expect, what to look for and how to communicate this to the patient effectively to help guide them through recovery, and of course, how to communicate deviations from this course to your team.

Knowing how a patient’s course and post-operative outcomes deviated from the expected trajectory will help you to anticipate what their needs will be at discharge, but also what obstacles they might face after discharge. In one study, the concept of a “Discharge Time Out” is suggested as a means to ensure that the surgical intern has addressed all of these concerns ( Mohta et al. 2012).

In a large randomized control trial, Better Outcomes for Older Adults through Safe Transitions (Project BOOST), preparing patients to successfully address situations after discharge was found to reduce 30-day readmission rates and improve hospital and primary care provider communication and collaboration ( Enderlin et al. 2013; Williams et al. 2014). There should be clear verbal communication between the team and the patient about the details of the hospitalization, including exactly what surgery was performed, what the post-operative goals of treatment and management are, where patients are in their recovery process, and any deviations in the expected course. These details should be communicated clearly in the discharge summary for other providers. If a patient has a prolonged hospitalization it can be difficult to retroactively review the chart for all the critical details. It is helpful to chronologically document the hospital course along the way and update as events occur.

**5. *Determine and confirm the discharge plan***. In the patient and caregiver advocacy literature, the following steps encompass “discharge planning” ( Family Caregiver Alliance 2009). Establishing expectations early on can go a long way in soothing anxiety and getting buy in from the patient and the family during the hospitalization and beyond. Give patients and caregivers an overall sense of what you need to accomplish and how they can help to ensure a safe discharge.

a. Patient evaluation by qualified personnel. This is accomplished during daily rounds by the resident team and discussions with the attending. Additionally, other consulting services, such as other medical teams, physical therapy (PT), occupational therapy (OT), wound and ostomy care teams, and nutrition, should all evaluate the patient when necessary. Their evaluation will help determine post-discharge care needs.

b. Discussion with the patient or [their] representative. Just as you discuss aspects of inpatient care decisions with the patient, you must discuss the details of discharge with each patient, and caregiver, when appropriate. They must understand what the recommendations are, accept them and be informed as to how they will be implemented at the time of discharge.

c. Planning for homecoming or transfer to another care facility. This is what we typically consider disposition planning. Working with the case manager, nursing staff, patient, and caregiver, can help determine what level of care is appropriate for the patient after discharge.

d. Determining support needs, particularly whether caregiver training will be sufficient or whether other support will be needed. With input from the primary team, PT/OT, wound care nurses, ostomy nurses and other ancillary staff, we can have a very comprehensive picture of what we think the patient needs. The next step is to determine whether or not the patient and/or their caregiver can perform their activities of daily living after surgery. The caregiver can provide valuable information about the patient’s capacity for functional recovery and should be included in discharge planning ( Family Caregiver Alliance 2009).

e. Referrals to a home care agency and/or appropriate support organizations in the community.

f. Arranging for follow-up appointments or tests ( Family Caregiver Alliance 2009). Patients should always leave with appointment dates, times, and contact information in hand. These should be printed and easy to locate on the discharge instructions.

***6. Collaborate and use your hospital resources**.* Numerous providers are involved in the process of discharging a patient. In a survey of internal medicine residents, there was substantial disagreement over one third of the discharge responsibilities ( Card et al. 2014). This highlights the importance of defining roles and responsibilities, as well as expected time frames and deadlines for each member of the team. It has been shown that daily multidisciplinary discharge rounds with representatives from case management, floor nurses, surgical team, and other core services such as PT/OT, can decrease length of stay and improve coordination of care ( Shardien 1997; Dutton et al. 2003; Haan et al. 2007). While your individual hospital workflow may be slightly different, at our institution the following providers work together in the transition period:

a. *Surgical intern.* During daily rounds, the intern communicates with their chief resident and attending to discuss anticipated discharge timing and needs. Typically, the intern communicates this information back to the patient and the rest of the care team. On the days leading up to discharge and the day of discharge, the intern completes the medication reconciliation including writing any new prescriptions, reviews any home service needs and assures that all orders/instructions are included where necessary, writes patient discharge instructions and a discharge summary, and places the discharge order.

b. *Advanced Practice Providers.* At our institution, the APPs provide month to month continuity on most surgical services. Their role in discharge may range from direct discharge care coordination and the provision of discharge orders and notes, to a more supervisory role in the discharge process.

c. *Other consulting services.* One concern that arises in discharging patients is the potential lack of communication with other consulting services. For example, in our institutional experience, if a patient has a closed suction drain placed by interventional radiology, they may not have accurate or up to date discharge instructions for management of this drain at home. It is critical that all teams caring for the patient have provided timely input at the time of discharge.

d. *Floor nurse.* At our hospital, the floor nurse is typically the last care team member that the patient sees before leaving the hospital. They provide patient education based on what is written into the discharge instructions. They also have the opportunity to bring up any new patient concerns or questions up until the moment the patient is transported off the ward.

e. *Case manager.* The case manager works closely with patients and families to discuss care needs after discharge. With close daily communication, the case manager and surgical intern ensure that the patient has the required resources to promote uneventfully recovery at their targeted disposition.

f. *Ancillary teams.* Our patients frequently require additional physical or occupational therapy, or wound and ostomy care during their hospitalization. Patients are typically provided specific instructions for their care, and ideally the ability to demonstrate competency of the desired task. It is important to incorporate these patient-centered orders in their discharge instructions.

g. *Outpatient nurse navigator.* When considering the predischarge, bridge, and postdischarge framework to reduce readmissions, utilizing the outpatient staff to help coordinate postdischarge care, can help patients transition to life at home after surgery.

***7. Don’t reinvent the wheel.*** While the discharge summary is a very critical document, and should be not be an empty template of pre-formatted electronic health data, rubrics exist to ensure that necessary information is included. The Joint Commission released a detailed list of the basic items needs for a discharge summary ( Kind & Smith 2008). Ensure that these details are accurately and efficiently documented every time. Creating a widely used template with easy to input variables allows for standardization of the process and can improve workflow among residents when appropriate feedback is given ( Showalter et al. 2011; Bischoff et al. 2013; Soong et al. 2016; Black & Colford 2017). We have included examples from our own surgical experience in Table 1.

**Table 1.** Joint Commission (JCO) Recommendations for Discharge Summaries with corresponding surgical relevance and examples.

JCO Recommendation	Surgical Relevance
Reason for hospitalization	- Chief complaint: Why did the patient require hospitalization? - History of present illness: Was this an emergency room admission, an admission for elective surgery or a readmission? If the patient had surgery, what was the indication? Example - The patient may have been admitted for failure to thrive due to inflammatory bowel disease, but they went on to have a total abdominal colectomy due to their toxic megacolon.
Significant findings	- Primary diagnosis: This is the main reason for hospitalization, usually the same reason for surgery. - Secondary diagnoses: Include both hospital and surgical significant findings, and any issues that complicated their hospital course. - Use physiciancentric language. Example - A patient’s primary diagnosis might be acute cholecystitis, but their secondary diagnses might include congestive heart failure (CHF) and prolonged ileus if these things occurred and needed to be managed during the hospitalization.
Procedures and treatments provided	- Procedures: Ensure accuracy, use operative note for clarification and avoid abbreviations and acronyms. - Example - Hand-assisted laparoscopic total abdominal colectomy on 8/18/18. - Hospital course: What would you want to know if you saw this patient for follow up in the clinic or in the emergency room? Example - For routine patients, the hospital course may simply explain our typical post-operative recovery. For complex patients, this may be organized in either a problem-based style or chronologically, include major events, consultations and ongoing issues, but try not to include too many resolved issues and unnecessary details.
Patient’s discharge condition	- This section is often overlooked or simply written as stable, improving, worse, or deceased. - Describe patient’s current functional status and cognitive capacity - Explain relative to baseline - May include rational for postdischarge care services Example - Patient is recovering well after surgery and back to baseline with respect to activities of daily living.
Patient and family instructions	- Address questions that a patient might ask or issues they will likely encounter at home. - Use patientcentric language - Deliver at healthliteracy level Example - Include activity restrictions, wound care instructions, discharge medication changes, dietary orders, specific instructions for any new devices (e.g. drains, ostomies, etc.), plans for medical/surgical follow-up.
Attending physician’s signature	- Chart signed by rounding attending surgeon on day of discharge - Discharge summary should ALSO include name of operating surgeon on record

**8. *Write for your intended audience.*** The discharge summary is a medical handoff, as mentioned in *Tip #1.* With the exception of the section for patient/caregiver instructions, this document should be geared toward medical providers. In a qualitative study of an interprofessional team of healthcare providers receiving discharge summaries from orthopedic surgery patients, discharge summaries were frequently found to be inadequate, inaccurate, and non-physician centric. This limited their utility in preventing patient harm in the transition period ( Soong et al. 2016). In non-surgical fields, the primary target of the discharge summary is the primary care physician who will take charge of further outpatient management of chronic illness ( Wimsett et al. 2014). In surgical fields, the discharge summary has a number of potential audiences beyond the PCP. For example, a future surgeon may read the discharge summary to determine what type of surgery this patient had and if there were any complications. Furthermore, stakeholders may include administrators who will read this document to determine the appropriate complexity of patient, which determines the diagnosis related group, or DRG, which affects reimbursements ( Centers for Medicare and Medicaid Services 2016). Additionally, if the patient is discharged to a skilled nursing facility, this document will help provide a template for future care needs. Portions of this document will also be available to the patient and caregivers.

**9. *Write patient-centered, health literate discharge instructions.*** Up to 60% of surgical patients have inadequate health literacy ( De Oliveira et al. 2015). Additionally, in the bariatric surgery population, achieving a lower level of education increases risk of readmission ( Mahoney et al. 2018). Health literacy is notoriously difficult to assess; even well-educated patients express difficulty understanding their medical condition or written hospital materials. For this reason, it can be advantageous to make use of the following resources:

a. NIH Plain Language Seminar - This resource will help clarify all of your written work, from discharge summaries aimed at patients and caregivers, memos to colleagues, or scholarly products ( National Institutes of Health 2016).

b. Office of Disease Prevention and Health Promotion provides valuable links and activities regarding health literacy and health communication ( Office of Disease Prevention and Health Promotion 2016).

c. Identify local resources, such as your hospital librarian, who may be able to assist you in determining whether or not your discharge materials are patient-friendly and understandable.

**10. *Educate the patient and their caregivers.*** Take every opportunity that you can throughout the hospitalization to reinforce necessary principles for success once at home. The discharge instructions (Tip #9), are an excellent tool to reinforce educational materials. It is important to assess the patient’s understanding of educational materials and discharge instructions. This assessment can be done using the teach-back method ( Agency for Healthcare Research and Quality 2017). Notably, in a survey of heart failure patients, those patients with non-perfect understanding of their discharge instructions were more likely to be readmitted, (p=0.044) ( Regalbuto et al. 2014). Additionally, a patient’s reported preparedness for discharge and self-efficacy, or ability to manage postoperative needs, is widely variable and can affect postoperative healthcare utilization ( Leppin et al. 2014; Mixon et al. 2016).

**Figure 1.**
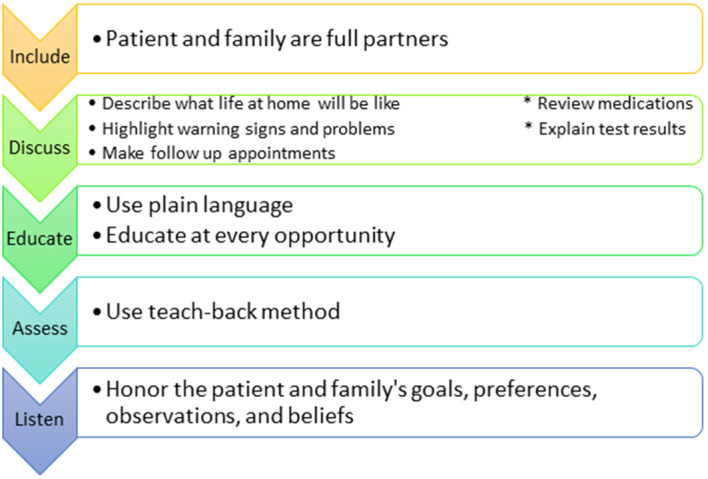
Agency of Healthcare Research and Quality IDEAL Framework for the discharge process.

**11**. ***Use the IDEAL Framework ( Agency for Healthcare Research and Quality 2017).*** AHRQ developed an interprofessional framework to reduce adverse events and improve patient safety. While the above tips can guide the surgical intern through planning and execution of a successful discharge summary, this framework reinforces the overarching goals of the discharge process and reminds us that the patient, their understanding, and their readiness is at the center of it all. We have created a visual representation of this framework in Figure 1.

**12. *Answer all questions in person.*** Make yourself available to the patient to answer any final questions prior to discharge. Also ensure availability to answer questions that may arise at home and provide reliable contact information. Being present and engaged improves patient satisfaction, and allows patients to ask important questions that may reduce their unplanned high resource healthcare utilization.( Farberg et al. 2013; Jalilvand et al. 2016) Do not assume that someone else has already taught the patient what they need to know. Finally ensure the patient has the necessary resources, such as correct contact information, to manage any emerging healthcare needs once they get home.

## Conclusion

Frequently, the above tips are learned throughout the intern year through experiential learning. In providing training to interns regarding their role in the discharge process and the importance of the discharge summary in the discharge process, we may be able to mitigate the “July Effect.” The general surgery intern plays a critical role in preparing the patient for discharge and must learn to work with their entire team effectively in order to accomplish a safe discharge.

## Take Home Messages

• Readmissions are preventable adverse events after surgery.
• The discharge summary is a key tool for communication with patients and providers.
• An excellent discharge summary includes feedback from all stakeholders.
• An excellent discharge summary may prevent avoidable readmissions.

## Notes On Contributors

All of the authors of the paper work together as part of a quality improvement focus to reduce avoidable readmissions after gastrointestinal surgery, they developed these twelve tips to help guide intern discharge training. This study is funded by the University of North Carolina Institute of Healthcare Quality Improvement Clinical Scholars Program.

Dr. Stephanie Lumpkin is a fifth year general surgery resident at the University of North Carolina at Chapel Hill and currently a postdoctoral research fellow. Her research focuses on healthcare utilization after colon and rectal surgery. ORCID https://orcid.org/0000-0003-3011-8918

Dr. Ian Kratzke is a second year general surgery resident at the University of North Carolina at Chapel Hill with a focus on health outcomes research.

Dr. Meredith Duke is a minimally invasive and bariatric surgeon and an Assistant Professor at Vanderbilt University. Her research interests include quality improvement in bariatric and foregut patients.

Dr. Nicole Chaumont is colorectal surgeon and an Assistant Professor at the University of North Carolina at Chapel Hill. She serves as the leader for Patient Safety and Quality Improvement in the Division of GI Surgery and her research interests include healthcare outcomes and quality improvement in colorectal surgery patients.
