# Supplementary material for: Twelve Tips for Preparing a Surgical Discharge Summary: Enabling a Safe Discharge
Source: MedEdPublish (2016). 2019 Mar 5;8:39. [Version 1] doi: 10.15694/mep.2019.000039.1 (PMC10712596; doi:10.15694/mep.2019.000039.1)
Supplement: Peer review history

# Reviewer report: Reviewer response for version 1

**DOI:** 10.21956/mep.19778.r29719

**Published:** 2019-03-15

**Authors:** Leila Niemi-Murola

**Affiliations:** 1 University of Helsinki

This review has been migrated. The reviewer awarded 4 stars out of 5 Thank you for the opportunity to review this interesting manuscript. According to previous literature, giving and receiving a hand-over report is a practical procedure seldom mastered by newly graduated residents. There is always a risk of losing information and consequently, an unplanned re-admission may occur.The authors start with motivating the resident by clarifying the goal of perioperative patient care and the discharge as an important part of this process. Nowadays surgical patients are often elderly people, having multiple pre-existing health problems, aggravated by the surgical procedure. Thus, it is a good advice to start with the discharge in mind. It is also a good advice to remember who is the intended audience reading the discharge report – family physician and other members of healthcare personnel of course, but also the patient and his/her relatives. Thus, one should avoid Latin, abbreviations and medical jargon.This paper introduces a practical concept of a safe discharge and one can recommend it to both residents and their supervisors. These twelve tips are not self-evident but they should be discussed as a part of specialist education.

Reviewer Expertise:

NA

No decision status is available

# Reviewer report: Reviewer response for version 1

**DOI:** 10.21956/mep.19778.r29720

**Published:** 2019-03-11

**Authors:** Julie Hunt

**Affiliations:** 1 Lincoln Memorial University

This review has been migrated. The reviewer awarded 4 stars out of 5 The focus of this 12 Tips document is to provide information to surgical interns about how and when to construct and deliver a successful surgical discharge. The authors provide sufficient justification for the importance of this document in terms of reduced hospital readmissions and post-operative complications. The paper does a good job of defining the different parties' motivations and interests during the post-operative period, and how these play a guiding role for what is included in the discharge document. The authors point out what ancillary services and collaborators should be involved in crafting the discharge plan without making assumptions about how that system works in every hospital. This paper would be a valuable read for incoming surgical interns and those responsible for training them.

Reviewer Expertise:

NA

No decision status is available
